# Evolution of Concepts: Can Personalized Hip Arthroplasty Improve Joint Stability?

**DOI:** 10.3390/jcm12093324

**Published:** 2023-05-07

**Authors:** Sivan Sivaloganathan, William G. Blakeney, Charles Rivière, Pascal-André Vendittoli

**Affiliations:** 1 Imperial College Healthcare NHS Trust, London W2 1NY, UK; 2 Department of Orthopedic Surgery, Royal Perth Hospital, Perth, WA 6000, Australia; blakeney@gmail.com; 3 Clinique de Sport Bordeaux-Mérignac, Bordeaux Arthroplasty Research Institute, Personalized Arthroplasty Society, 33700 Mérignac, France; c.riviere@imperial.ac.uk; 4 Hôpital Maisonneuve-Rosemont, Clinique Orthopédique Duval, Personalized Arthroplasty Society, Montréal, QC H1T 2M4, Canada

**Keywords:** hip arthroplasty, personalized arthroplasty, kinematic total hip replacement, kinematic, hip dislocation

## Abstract

Hip arthroplasty procedures are successful and reproducible. However, within the first two post-operative years, hip dislocations are the most common cause for revisions. This is despite the majority of the dislocations having the acetabular component within what is described as the ‘safe zone’. The limitations of such boundaries do not take into account the variability of individual hip anatomy and functional pelvic orientation that exist. An alternative concept to address hip instability and improve overall outcomes is functional acetabular orientation. In this review article, we discuss the evolution of concepts, particularly the kinematic alignment technique for hip arthroplasty and the use of large-diameter heads to understand why total hip arthroplasty dislocations occur and how to prevent them.

## 1. Introduction

The hip joint constitutes an intricate relationship between anatomy and biomechanics. It is this relationship that enables stable movements. Despite the complexity of attempting to reproduce native hip function, total hip arthroplasty (THA) is one of the most successful and reproducible procedures in orthopaedic surgery. The foundation for this success is multifactorial. Hip arthroplasty has become less invasive, implants are now more resistant to wear and loosening, and peri-operative management is increasingly more focused on patient optimization [1,2].

In this review article, we evaluate the history of acetabular cup placement in relation to hip dislocation. We introduce the concepts that underpin personalized hip arthroplasty from both the kinematic alignment technique and the use of more forgiving implants such as large-diameter bearings. The scope of this article is not to focus on the multivariant causes that can lead to hip dislocation; the scope focuses on the role of functional acetabular orientation in relation to the spinopelvic parameters that when better understood can influence acetabular cup placement in addition to cup design selection. Literature databases were searched between 31 January 2005 and 30 November 2022. The primary search was conducted using the electronic databases MEDLINE, EMBASE, PubMed, and Google Scholar. The following inclusion criteria were applied to the primary search: publications in English in peer-reviewed journal reports on clinical outcomes, all hip pathologies were included, and no restriction to the type of study. The exclusion criteria applied included: non-English publications. A secondary search was conducted using review articles and Google Scholar.

The UK National Joint Registry (NJR) substantiates the ongoing success of primary THA. The data illustrates a decline in revision rates for THA between 2008 and 2020. The declining trend in the rate of revisions is a success story; however, revisions due to dislocations continue to challenge further improvements in outcomes. For a primary THA performed in 2010, the 10-year revision rate is 3.7% (95% CI 3.5–3.8%) [3]. Dargel et al. state that dislocations following a primary THA vary annually between 0.2% and 10%; 2% of dislocations occur within the first 2 years [4]. Other studies suggest a variable range of up to 5% of primary THA results in dislocation [5,6,7,8,9]. Within the first two post-operative years, dislocations are the most common cause of revision surgery [10]; 50% of dislocations occur within the first 3 months of the index procedure, and more than 75% occur within the first year [11].

Some hip surgeons have pivoted increasingly towards personalized hip arthroplasty to address the complications associated with conventional primary THA. However, personalized hip arthroplasty is not one single technique, and there are a number of proposals to address the challenges of reducing complication rates while optimizing clinical outcomes (function, perception, and longevity). Personalized hip replacement, among other aspects, involves the individualization of components’ position/orientation, trying to match each patient’s specific hip anatomy and lumbopelvic kinematics (publicized as the kinematic alignment (KA) technique), and utilization of more anatomical/forgiving implants: large-diameter bearing, fixed (LDH) or with dual mobility (DM).

It is the KA technique that is primarily focused on functional acetabular orientation to optimize cup position and accommodate the natural and pathologic variations in the hip joint. The two key pillars of the KA technique are the restoration of the native hip anatomy (unless abnormal) and cup design selection and adjustment of its orientation (functional cup positioning) which depends on the individual spine–hip relationship. KA technique is one of the concepts that is driving the evolution of THA. This manuscript aims to review these concepts, among others, to prevent hip instability and provide patients with an unrestricted hip range of motion after their hip replacement.

## 2. Traditional Concepts

### The Safe Zone Concept and Dislocations: The Rationale for Change

Maintaining hip stability and restoring normal joint biomechanics in THA is a balance between the positioning of implants and the resultant soft tissue tension. For decades, there has been a focus on implant position within a determined safe zone. The Lewinnek’s safe zone is considered to be an acetabular cup inclination of 40° ± 10° and an acetabular anteversion of 15° ± 10° [12]. However, current evidence suggests that even in implants positioned within this safe zone, dislocations still occur [13,14]. A study by Abdel et al. analysed 9784 primary THAs and concluded that the majority of dislocations were in hips within those target values [11]. Such boundaries do not take into account the variability of individual hip anatomy and pelvic orientation that exist. An alternative concept to address hip instability and improve overall outcomes is functional acetabular orientation.

Functional acetabular orientation is determined by the relationship between the lumbar spine position and the pelvic position (orientation of the acetabulum) [15]. This concept offers an explanation as to why some patients with standard cup orientation dislocate, whilst others with abnormal cup orientation do not [16]. A clinical example of spinopelvic-driven pathology is the higher dislocation rate after THA in patients with adult spinal stiffness, than in healthy controls (8% vs. 1.5%) [17,18,19,20,21,22].

## 3. Evolution of Concepts: Spinopelvic Parameters

### Hip-Spine Alignment: The Spinopelvic Parameters and the KA Technique

The KA technique for THA is a meticulously planned approach. Medical history taking and clinical examination are the initial steps of identifying hip and spine conditions that may be associated with hip osteoarthritis (OA). This is followed by radiological imaging (standard radiographs or EOS) that include supine and standing anteroposterior (AP) pelvic views. The supine AP pelvis is the gold standard for estimating frontal hip anatomy and leg length. The standing AP allows a review of the functional position—pelvic obliquity (secondary to spinal deformity or leg-length discrepancy) and acetabular orientation. Both the supine/standing AP images allow the evaluation of functional positions. A cross-table lateral radiograph with the patient in the supine position with the leg internally rotated at 15° completes the imaging by delivering an estimate of the functional combined anteversion in the supine position [23].

**Figure 1:**
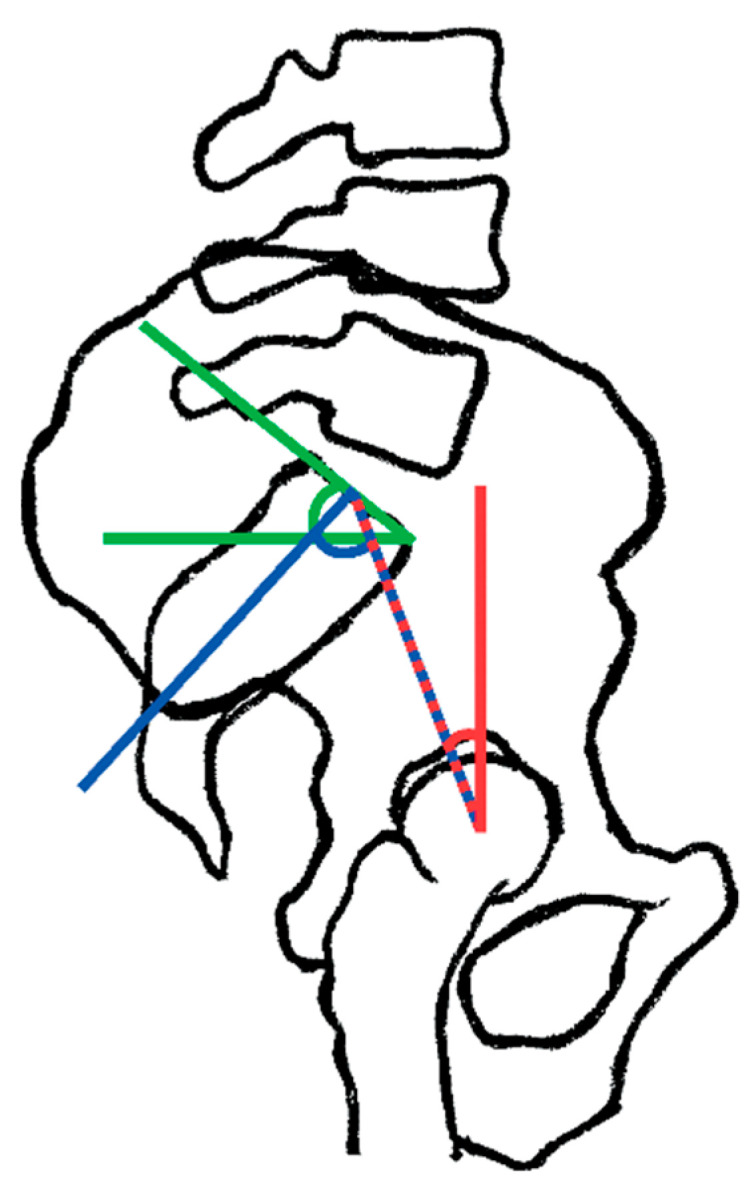
The key spinopelvic parameters: pelvic tilt (red); pelvic incidence (blue); sacral slope (green) (33).

**Figure 2:**
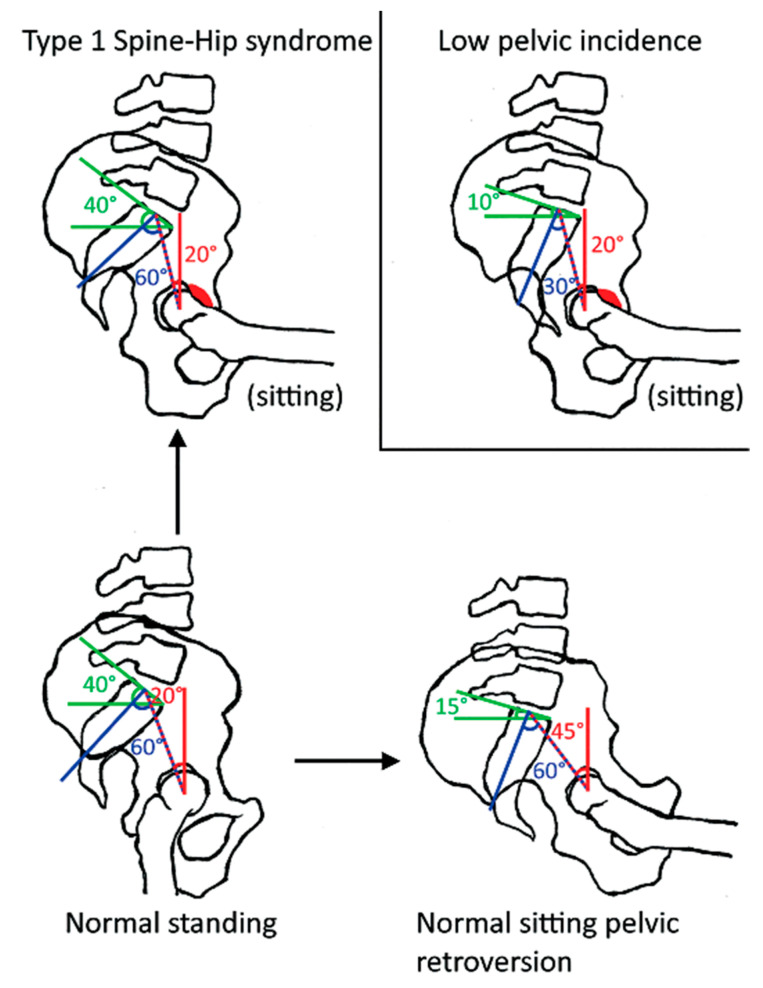
A flexible lumbopelvic complex and flexible hips reduce the risk of femoral-acetabular impingement (**bottom** images). Abnormal spine–hip relationship can lead to femoral-acetabular impingement (red) as a result of type 1 spine-hip syndrome (**top left**) or low pelvic incidence (**top right**) (33).

**Figure 3:**
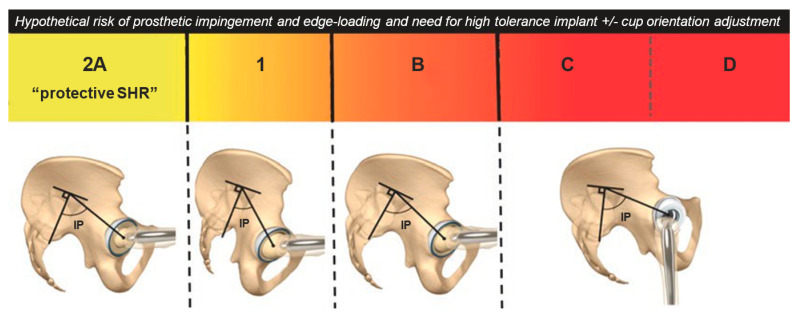
Simplified Bordeaux classification of spine–hip relationship: the risk of dislocation increases from left to right with an increasing need for cup adjustment to mitigate this risk (15).

Radiographs for the KA technique (lateral images) were taken in the standing and deep sitting positions (with back in flexion) to better estimate the flexibility of the lumbar spine. These are used to assess the spinopelvic parameters (Figure 1) that are divided into:

(1) Positional parameters, such as sacral slope (SS) (Figure 1), pelvic tilt (PT) (Figure 2), pelvic-femoral angle (PFA), and lumbar lordosis (LL).
(2) The anatomical/morphological parameter is the pelvic incidence (PI) (Figure 3).

The interaction between these spinopelvic parameters determines the movement between the spine and the hip in the sagittal plane. Pelvic incidence (range of 35° to 85°) is a constant that does not change with posture, movement, or time; it is the sum of SS and PT. A greater lumbar lordosis is reflected by a larger PI, whereas a flat back is reflected by a smaller PI. The PI ultimately reflects the ability of the individual to compensate for sagittal imbalance through pelvic retroversion [24,25].

The pelvic tilt is a guide to the orientation of the pelvis. Changing from a standing to a sitting position results in more pelvic retroversion (the favoured motion when sitting) which is illustrated by an increasing PT (20° to 45°). The sacral slope is a reflection of the position of the lumbar spine. Sitting from a standing position results in a decrease in SS (40° to 15°).

The PFA can be used to measure the position of the femur relative to the pelvis when standing (180°) and sitting (125°). This along with the lumbar lordosis is key to calculating overall spinopelvic movement (sagittal flexion arc, SLA) and hence determining if the spinopelvic movement is within a normal range, or if most of the patient’s spinopelvic movement comes from either the hip (hip user) or spine (spine user). For the KA technique, aided by the EOS imaging system, the key parameters used are the sagittal vertical axis (distance from the plumb line from the centre of the C7 (C7PL) to the posterior edge of the upper sacral endplate surface), PI, and change in LL (standing to sitting) [15].

## 4. Clinical Application of Spinopelvic Parameters

### 4.1. Practical Application of Spinopelvic Parameters: Hip Users vs. Spine Users

Moving from a standing to a sitting position combines three movements in the lumbopelvic complex (LPC); this includes hip flexion, a reduction in lumbar lordosis (decrease in LL), and a posterior tilt (retroversion, increase in PT) of the pelvis [24,26]. Retroversion of the pelvis is a roll-back mechanism, which allows a change of position that prevents impingement of the femoral neck against the anterosuperior rim of the acetabulum. A reduced pelvic roll-back in the sitting position requires a compensatory increase in femoral flexion to allow sitting. This deficit in retroversion results in reduced coverage of the femoral head posteriorly. In a prosthetic hip, this leads to an increased risk of anterior impingement and posterior dislocation [26,27].

Spinopelvic movements are the difference between the standing and sitting positions for each of the spinopelvic parameters. The sagittal flexion arc (SFA) (the whole spinopelvic movement) is the sum of ∆LL and ∆pelvic femoral angle (PFA). The hip user index is a percentage that illustrates sagittal hip flexion relative to the overall SFA when moving from the standing to the sitting position [28,29]:

Hip user index = ΔPFA/SFA × 100% [29].

A high hip user index (>80%) indicates a greater contribution of hip movement to the overall sagittal movement. Likewise, in a low hip user index (spine user), the compensatory movement to sit down takes place in the lumbar spine. Any pathology that affects the hip range of motion could increase the risk of impingement, dislocation, and edge loading in patients with THA [29,30].

#### 4.1.1. Spinopelvic Pathology Overview

A normal spine–hip relationship is the co-existence of a flexible lumbopelvic complex and flexible hips. If either of these is stiff, a compensatory mechanism is required by the other. This is defined as a spine-hip syndrome (SHS) or hip-spine syndrome (HSS), depending on whether the primary pathological structure is the spine or the hip, respectively [31,32].

#### 4.1.2. Spine-Hip Syndrome (Figure 2)

In degenerative spines, there is a loss of lumbar lordosis. This results in a pelvic retroversion. The patient often compensates for this by extending the hips. Over time this fails, and the patient hyperextends their neck to keep a horizontal sightline. These patients are at risk of under-coverage of the femoral head anteriorly and posterior impingement. When these patients undergo THA, they are at risk of complications such as anterosuperior edge loading/posterior prosthetic impingement, and anterior instability of the prosthesis when standing [33].

In patients with muscle imbalance, there is often not enough retroversion when sitting. This results in abnormal functional acetabular orientation in the sitting position causing an over-coverage of the femoral head and anterior impingement. This can lead to osteoarthritis in the native hip and posterior instability/posterior edge loading in THAs. The diagnosis is made on lateral imaging showing <10 degrees change in pelvic retroversion between standing and deep sitting. A small PI of <35 degrees can increase the risk of edge loading and instability. As a general rule, these patients are constitutionally hip users. A large PI results in less hip movement (low hip users or spine users) [33].

### 4.2. Hip-Spine Syndrome

Hip osteoarthritis makes the hip stiffer. The lumbopelvic complex will compensate for the reduced hip movement by increasing lumbar lordosis. This eventually results in degeneration in the spine which results in chronic back pain [33].

The Bordeaux classification of the spine–hip relationship (Figure 3), devised by Rivière et al. in 2017 [16,34], is currently the most complete and comprehensive classification system. It describes the relationship between the hips and the spine and allows for the categorizing of the risk of primary THA impingement or dislocation [35].

There are three broad risk categories in the simplified Bordeaux classification:

A (low-risk);

Idiopathic B and C (moderate risk);

D (high risk).

Category A (in lumbopelvic complex type 1 (PI < 40°) or type 2 (PI > 40°)) refers to a healthy lumbopelvic complex with >10° of retroversion when the patient is sitting. In category B, there is a stiffer lumbopelvic complex with <10° of retroversion when the patient is sitting, but the standing posture is normal; these patients have an increased risk of posterior edge loading, anterior impingement, and posterior dislocation when sitting or squatting. Categories C and D represent degenerative spinal changes in which there is both a stiffer lumbopelvic complex and a constant pelvic retroversion when the patient is standing while sagittally balanced (C, compensated) or imbalanced (D, decompensation) [15]. From a practical perspective, there are two primary pathological outcomes to be aware of when performing a THA. The pelvis does not retrovert enough on sitting (Larry Dorr’s stuck standing) and the pelvis is in a chronically retroverted position on standing (Larry Dorr’s stuck sitting). In both cases, they affect the acetabular orientation in sitting (type 1) and standing (type 2).

## 5. Operative Planning

### 5.1. Impact of Spinopelvic Parameters on Pre-Operative Planning: Implant Position and Implant Choice

Inadequate congruency between articulating implant surfaces in THA can lead to edge loading. An abnormal hip–spine relationship increases the risk of edge loading. While anterosuperior edge loading can occur in extension, posterior edge loading can occur in flexion. By factoring in functional acetabular orientation, the risk of edge loading can be minimized [36].

The KA alignment technique for THA allows for targeted orientation of the acetabular component. It is a patient-specific approach that factors both constitutional acetabular anatomy (for version) and pelvic kinematics. The KA-THA technique builds on the work by Legaye et al. [36,37]. While the technique can be performed using manual instrumentation, intra-operative precision tools (robotic/navigation systems or patient-specific instruments) may be of value to assist in achieving the target placement.

Determining cup adjustment is a core feature of the KA technique as it is the kinematic cup adjustment that aids in compensating for any abnormal spine–hip relationship (SHR). Planning a radiographic cup inclination below 45° is required to reduce superior edge loading when standing. KA-THA cup anteversion is based on the transverse acetabular ligament (TAL) orientation, not the anterior pelvic plane. The adjustment to cup orientation is required when more forgiving implants (LDH) cannot sufficiently compensate for poor functional acetabular orientation; as the risk increases in the Bordeaux category, this need for adjustment increases [15]. The practical application of cup adjustments is based on observational data. The average posterior tilt change from standing to sitting is 20° [38,39]. Every 10° of pelvic tilt is associated with a 3° inclination and 7° anteversion in cup orientation [40]. Defining the SHR starts with a clinical examination. If there is evidence of a fixed flexion deformity in the hip (Thomas test), this is suggestive of a significant sagittal imbalance (SHR D—high risk of dislocation). If there is no evidence of a sagittal imbalance, a standing lateral lumbopelvic radiograph is performed. Here, if there is a PI-LL mismatch, the patient is SHR C. If there is no PI-LL mismatch, then a comparison of lateral standing and sitting lumbopelvic radiographs is performed to assess if delta SS < 10° (SHR B) or if delta SS > 10° with proportional delta LL (SHR A).

For SHRs that are low risk (SHR A), the use of a large-diameter bearing is of less importance and the anatomic cup version (TAL) is used as a reference. For high-risk SHRs, implants that reduce the risk of dislocation (LDH) can be used along with a cup version based on the TAL. For SHR B, there should be an increase in cup anteversion by 3.5° for every 10° deficit in pelvic retroversion when sitting. SHR D requires a reduction in cup anteversion by 3.5° for every 10° excessive retroversion when the patient is standing. For the high-risk THAs, we would recommend using a large-diameter bearing, fixed (LDH cups) or mobile (dual mobility) depending on a patient’s activity and life expectancy [15,16,35,41].

### 5.2. Additional Benefits of Optimized Bearing Orientation

(i) Bearing surfaces

Highly cross-linked polyethylene liners are preferred over ceramic-on-ceramic bearings in abnormal lumbopelvic kinematics. Though ceramic liners have excellent wear properties, there is a higher risk of liner fracture associated with impingement/edge loading in the standing position, with these being more common with abnormal lumbopelvic kinematics. ‘Squeaking’ can also be present in ceramic-on-ceramic liners where there is edge loading/micro-separation. Generally, due to these risks associated with the malpositioning of the cup, ceramic-on-ceramic liners are avoided in high-risk patients [42].

(ii) Stem sizing, design, and fixation

Prevention of impingement begins with an accurate reconstruction of hip length and offset. Native femoral anteversion can range between 30° (anteversion) and −15° (retroversion) [43]. Cemented stems can be rotated within the femoral bone to achieve the intended version, whereas uncemented stems require a press-fit. As a consequence, there is often less margin to adjust the stem anteversion in cementless designs [42].

### 5.3. Functional Alignment, Limits of the Concept

There are limitations to the concept of functional alignment. Ageing can alter the relationship between the hip and spine as a result of degenerative changes. This results in the initial implant orientation becoming less optimal. The use of LDH implants mitigates these changes. The spine–hip relationship can also be a result of the hip-spine syndrome and may change following arthroplasty (e.g., when removing the mechanical block, osteophytes).

This concept is grounded in the fact that the current widely practised techniques change the joint anatomy, the head-neck offset, and the joint diameter (small bearings < 36 mm). An alternative/additional option to consider is the use of LDH implants (increasing the head-neck offset) that are more forgiving and allow a more physiological range of motion [44,45].

### 5.4. Large Head Diameter (LDH) Implants

LDH THA is defined by a bearing diameter >36 mm and is available in two constructs: fixed bearing or dual mobility [1]. In addition to the greater jump distance, combining an LDH and a 12/14 femoral neck optimizes the femoral head-neck offset, offering an implant that delivers an impingement-free range of motion and a significantly reduced risk of dislocation. Such supraphysiologic stability can compensate for a patient’s anatomical variations, abnormal spinopelvic mobility, and surgical imprecisions. Using LDH implants (fixed or dual mobility) was shown to significantly reduce the dislocation rate; a study by Zijlstra et al. evaluated 160,000 THAs from the Dutch arthroplasty register and noted that the risk of dislocation was higher in 22–28 mm heads (1.1%) when compared to >36 mm heads (0.5%) [46,47,48]. In our case series of more than 4000 LDH THA with ceramic bearing (since 2011) and without ROM or activity restriction post-operatively, we encountered 4 single and 1 recurrent episode of atraumatic dislocation (0.13%) [49].

Additionally, LDH ceramic-on-ceramic bearing with a small clearance exerts a high suction force, which provides greater hip micro-stability. With appropriate biomechanical reconstruction, LDH THA can restore normal gait parameters [50]. Less influenced by the risk of instability, surgeons using LDH THA can better optimize a patient’s leg length and femoral offset. This results in unrestricted activities and a high patient satisfaction score [1].

### 5.5. Advances in Precision Technology: Robot-Assisted THA

Precision tools have become available in recent years. An example of this includes the robotic-assisted THA. The CT-based robotic system stores and evaluates CT imaging of the pelvis. Sagittal radiographs in both the standing and sitting positions are performed for lumbopelvic parameters. The values obtained are utilized in the pre-planning system along with pelvic tilt, allowing evaluation of the lumbopelvic relationship. This also allows for optimal inclination/version to be simulated prior to implant placement. The added benefit of these systems is that they can allow for a virtual range of motion impingement detection. Where there is evidence of impingement, the inclination/version can be adjusted. The use of the robotic system can allow for improvement in the placement of the cup—an important parameter that plays a significant role in the long-term success of THA. However, whether the radiographic improvements observed will translate into reductions in component impingement, acetabular wear, prosthetic dislocations, or longevity requires a longer-term follow-up [51].

### 5.6. Clinical Outcomes: The Evidence So Far

Published studies with clinical results using this personalized approach are limited. There is variability in the definition of personalized arthroplasty. This ranges from kinematic alignment (taking into consideration the constitutional hip anatomy and the spine alignment and flexibility), the use of robotics for precise placement of the hip centre of rotation, to using more anatomic implants (e.g., large-diameter heads to restore normal biomechanical function).

Riviere et al. published a retrospective study comparing kinematic alignment against conventional alignment techniques for THA. The study concludes that the KA technique resulted in a more natural restoration of the prosthetic hip centre of rotation with a lower change in pre- to post-operative horizontal acetabular offset. The KA technique also resulted in a higher cup anteversion but comparable cup inclination. It also found similar excellent functional outcomes and similar patient satisfaction scores with minimal complications [52]. In another study by Blakeney et al., using LDH THA without precision tools on 276 hips (246 patients) at a mean of 67 months (48 to 79) postoperatively, the patients had a Forgotten Joint Score of 88.5 (23 to 100) and no hip dislocation was reported. Such results are excellent, and the marginal gain is minimal [49].

## 6. Conclusions

Personalized hip arthroplasty offers an exciting approach to the management of hip arthritis. Applying the principles considered in this paper, hip surgeons can optimize implant orientation according to each patient’s anatomical variation and functional acetabular orientation (dictated by spinal alignment and kinematics). This may improve joint stability and reduce mechanical complications (dislocation and edge loading/prosthetic impingement). In addition to personalized implant orientation, using an LDH bearing (fixed or DM) enables a safe restoration of a larger range of hip anatomy and better compensates for poor functional acetabular orientation. The main goal of personalized joint replacement is to meet the patients’ objective of a ‘forgotten joint’ that delivers both unrestricted joint range of motion and activities. Although there is currently a paucity of evidence for clinical outcomes, the evolution of the concept is delivering a more refined approach to individual anatomy.

## Data Availability

Not applicable.
